# Increasing Hematopoietic Stem Cell Yield to Develop Mice with Human Immune Systems

**DOI:** 10.1155/2013/740892

**Published:** 2013-02-14

**Authors:** Juan-Carlos Biancotti, Terrence Town

**Affiliations:** ^1^Regenerative Medicine Institute, Cedars-Sinai Medical Center, Room 361, Steven Spielberg Building, 8700 Beverly Boulevard, Los Angeles, CA 90048, USA; ^2^Department of Biomedical Sciences, Cedars-Sinai Medical Center, Room 361, Steven Spielberg Building, 8700 Beverly Boulevard, Los Angeles, CA 90048, USA; ^3^David Geffen School of Medicine, University of California, Los Angeles, Los Angeles, CA 90048, USA

## Abstract

Hematopoietic stem cells (HSCs) are unique in their capacity to give rise to all mature cells of the immune system. For years, HSC transplantation has been used for treatment of genetic and neoplastic diseases of the hematopoietic and immune systems. The sourcing of HSCs from human umbilical cord blood has salient advantages over isolation from mobilized peripheral blood. However, poor sample yield has prompted development of methodologies to expand HSCs *ex vivo*. Cytokines, trophic factors, and small molecules have been variously used to promote survival and proliferation of HSCs in culture, whilst strategies to lower the concentration of inhibitors in the culture media have recently been applied to promote HSC expansion. In this paper, we outline strategies to expand HSCs *in vitro*, and to improve engraftment and reconstitution of human immune systems in immunocompromised mice. To the extent that these “humanized” mice are representative of the endogenous human immune system, they will be invaluable tools for both basic science and translational medicine.

## 1. Introduction

Hematopoietic stem cells (HSCs) were the first class of stem cells used for cell-based therapy in humans. Specifically, both autologous and allogeneic HSC transplantation (HSCT) have been practiced for decades to treat a variety of hematologic malignancies and congenital and autoimmune disorders [1, 2]. Fewer than 30% of patients requiring allogeneic HSCT have a histocompatible sibling, and it is exceedingly rare for patients to have an identical twin donor. Infectious complications and acute or chronic graft-versus-host disease (GVHD) remain the major obstacles affecting patient outcome after allogeneic HSCT [3]. Strikingly, GVHD occurs in approximately 20% to 50% of patients who receive stem cells from a human-leukocyte-antigen- (HLA-) identical sibling donor. Chances increase to 50–80% for those who receive stem cells from an HLA-mismatched sibling or even from an HLA-identical unrelated donor, while chronic GVHD occurs in less than 50% of long-term survivors [4]. Interestingly though, patients with acute GVHD have lower incidence of leukemia relapse, presumably owing to concurrent graft-versus-leukemia response [5]. This beneficial effect may not be limited to patients with leukemia, because something similar was observed in lymphoma patients that received allogeneic bone marrow transplantation [6].

Most commonly, HSCs are obtained by apheresis of adult peripheral blood after mobilization of bone marrow HSCs by granulocyte-colony stimulating factor (G-CSF) injections [7]. As an alternative, HSCs can be isolated from fresh or banked umbilical cord blood (CB) [8], which is highly enriched in HSCs compared to peripheral blood. Benefits of cord blood for transplantation include availability of banked samples, absence of risk to the donor, and low risk of transmitting infectious diseases. A specific advantage of cord blood over bone marrow-derived HSCs is reduced incidence of graft failure and acute or chronic GVHD, especially when cryopreserved CB is used for transplantation [9–11].

Despite having higher HSC concentration than peripheral blood, CB samples are insufficient to provide enough CD34^+^ cells for successful transplantations in adults and therefore require “pooling” samples from multiple donors. In addition to poor yield, shortage of HLA-matched cord blood samples has stimulated the development of methodologies to allow *ex vivo *HSC expansion. In principle, these methodologies would maintain self-renewal and inhibit differentiation during the course of expansion. The vast majority of strategies to expand HSCs *in vitro* have focused on regulation of stem cell renewal and survival of HSCs mediated by intrinsic factors (transcription factors and signaling molecules) and environmental cues (cytokines, chemokines, and adhesion molecules) [12]. 

An alternative application for HSCs has been to generate mice bearing human immune systems—so-called “humanized” mice. This experimental model was developed to address difficulties associated with studying human immune-related diseases in mice (this has been reviewed in [13–16]). Although a fully functional human immune system has not yet been achieved in the mouse, several strategies have been implemented with variable success. 

In this review, we consider various methodologies for maintaining HSCs for the purpose of reconstituting mice with human immune systems.

## 2. Mouse Models of Hematopoietic Stem Cell Engraftment

The development of chimeric mice bearing human immune system components provides a valuable tool to study human immune responses using small animals. In terms of disease biology, humanized mice can be used to study infection with human-specific pathogens, human autoimmune diseases, and human-specific immune responses in many contexts. These unique models can be created by engraftment of immunodeficient mice with human CD34^+^ HSCs. A crucial step towards the creation of immunodeficient mice that efficiently accept xenografts was the crossing of nonobese diabetic (NOD) and severe combined immunodeficient (SCID) mouse strains [17]. These NOD-SCID mice display T, B, and NK cell immunodeficiency, in addition to being deficient for macrophages and protein complement. These compound immune deficient mice enable increased chimerism upon HSC transplantation compared to SCID mice [15]. However, these animals have poor human T and B cell maturation, which has limited their use in immunology research. Targeting of cytokine receptors with IL-2R*β* monoclonal antibody prior to transplantation of human HSCs has allowed for even greater engraftment efficiency and human T cell development in the NOD-SCID mouse thymus [18]. Concurrently, new strains of mice deficient for the common cytokine receptor *γ*-chain (Il2r*γ*) have been generated. These include NODLtSz-SCID Il2r*γ* ^null/null^ (NSG; Il2r*γ* is completely null), NODShi-SCID Il2r*γ* ^−/−^ (NOG; the Il2r*γ* chain lacks the intracytoplasmic domain) [19–22], and BALB/c Rag2^−/−^ Il2r*γ* ^−/−^ mice (BRG) [23, 24]. These important immunocompromised mouse strains have become the most common vehicles for reconstitution of the human hematolymphoid system. Engraftment of CD34^+^ HSCs into these mice leads, under the right conditions, to differentiation and maintenance (for >6 months) of B and T lymphocytes, NK cells, dendritic cells, monocytes, erythrocytes, and platelets [20, 23, 24]. 

Myriad conditions contribute to the reconstitution success of engrafted immunodeficient mice. For example, the source of the HSCs plays an important role, with CD34^+^ HSCs derived from fetal liver or CB providing improved immune reconstitution compared to G-CSF-mobilized adult peripheral blood cells [25]. The age of the recipient immune compromised mouse is also critical, with neonatal recipient mice exhibiting enhanced engraftment compared to adults [26]. A third key factor is the genetic background of the recipient mouse strain. For example, NSG and BRG mice are equivalent in terms of generation of human B cells, dendritic cells, and platelets, whereas NSG mice are superior in supporting human T cell development [25, 26].

This salient difference on T cell development is based on a polymorphism in the gene encoding the signal-regulatory protein alpha (SIRP*α*) receptor [27]. SIRP*α* is a receptor expressed mainly in macrophages, granulocytes, and dendritic cells, but its ligand, CD47, is almost ubiquitously expressed. SIRP*α* binds to CD47 and generates an inhibitory signal to macrophages, which prevents phagocytosis of CD47-expressing cells. Mouse SIRP*α* interacts weakly with human CD47, with the upshot being phagocytosis and therefore rejection of transplanted human cells. However, NOD mice have a polymorphic allele of SIRP*α* that binds with high affinity to human CD47, preventing human cells from macrophage-mediated phagocytosis and leading to graft tolerance.

Although the presence of human cells can be detected in chimeric mice for 12 months, all hematopoietic subsets begin to decline around 6 months after transplantation [28, 29]. This effect is probably due to the inability of mouse cytokines to react with human receptors, leading to survival signal and trophic factor deprivation in transplanted human cells. One strategy to overcome this is supplementation with human cytokines; the concept is to create a more favorable immunologic environment for human cells within the mouse host. Another approach to transiently increasing hematopoietic cell lineages in humanized mice has been to inject recombinant proteins including interleukin (IL)-15 [30], IL-7 [31], B-cell activating factor [32], or hydrodynamic injection of a plasmid DNA mixture including IL-15 + Flt-3L and Flt-3L + granulocyte monocyte-CSF(GM-CSF) + IL-4 [33]. Human IL-7 has also been expressed in BRG mice by *in vivo* lentiviral gene delivery, and this led to stable but supraphysiological levels resulting in increased abundance of T cells [34]. Transgenic mice have also been used to stably increase expression of human cytokines. For example, forced expression of stem cell factor (SCF), GM-CSF, and IL-3 on the NOD-SCID mouse background (NS-SGM3) produced robust human hematopoietic reconstitution in blood, spleen, bone marrow, and liver and significantly increased myeloid cell numbers [35, 36]. Similarly, transgenic NSG mice expressing membrane-bound SCF exhibited a high degree of human CD45^+^ cell chimerism in irradiated [37] and nonirradiated [38] recipient pups.

**Figure 1:**
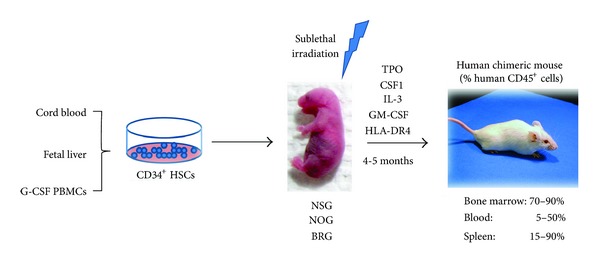
Generation of “humanized” mice. The reconstitution of the hematolymphoid compartment in immunocompromised mice is carried out by human CD34^+^ HSCs. Human HSCs can be isolated from umbilical cord blood (CB), fetal liver, or adult peripheral blood mononuclear cells (PBMCs) after mobilization of bone marrow HSCs by granulocyte-colony stimulating factor (G-CSF) injections. Although sublethally irradiated newborn NSG, NOG, or BRG immunocompromised mice are the most permissive recipients for engraftment of human CD34^+^ HSCs, exogenous supply of human cytokines or HLA class II transgenes creates a better environment for cell engraftment and improved development and function of the resultant differentiated immune cell lineages. Abbreviations used: BRG: BALB/c Rag2^−/−^ Il2r*γ* ^−/−^, CSF1: colony stimulating factor 1, GM-CSF: granulocyte-monocyte colony stimulating factor, HSCs: hematopoietic stem cells, IL-3: interleukin-3, NOG: NODShi-SCID Il2r*γ* ^−/−^, NSG: NODLtSz-SCID Il2r*γ* ^null/null^, TPO: thrombopoietin.

A more radical strategy has been to engineer a knock-in mouse in which the genes encoding mouse cytokines have been replaced by their human counterparts. Though laborious, this strategy has major advantages including stable expression of physiological levels of cytokines and localization to the right organ(s). Thus far, three mice have been reported, including one that expresses human thrombopoietin (TPO) [39], another expressing human CSF-1 [40], and an animal that expresses both human IL-3 and GM-CSF [41]. The TPO knock-in mice demonstrated improved engraftment of human CD34^+^ hematopoietic and progenitor cells, especially in bone marrow, and long-term maintenance of chimerism for over 6 months. Interestingly though, generation of the myelomonocytic lineage was particularly favored in these mice compared with lymphoid lineages. Mice expressing human CSF-1 displayed increased frequency and more efficient differentiation of human fetal liver-derived HSCs into monocytes/macrophages in various organs and increased functional properties, such as migration, phagocytosis and activation, and response to LPS. Transplanted IL-3/GM-CSF knock-in mice had no significant improvement in human hematopoietic engraftment, although enhanced reconstitution with human alveolar macrophages was reported [41]. This specific effect on the lung macrophage subset makes IL-3/GM-CSF knock-in mice a unique model to study the involvement of the immune system in human lung pathologies. Yet, iatrogenic events have been reported in some of these mouse strains. For example, the TPO knock-in strain developed thrombocytopenia [39], despite the fact that human TPO can support murine thrombopoiesis. This effect is likely owed to levels of human TPO expressed in this mouse that were ~10-fold lower than the endogenous murine TPO. In knock-in mice expressing IL-3/GM-CSF, nonengrafted mice developed pulmonary alveolar proteinosis caused by the absence of mouse GM-CSF [41].  Figure 1 summarizes various approaches to the design of humanized mice.

## 3. Human HLA Transgenic Mice

Humanized mice generated by transplantation of human HSCs have demonstrated long-term reconstitution and some degree of maturation of human T cells, evidenced by the presence of CD4/CD8 single-positive T cells in the spleen and peripheral blood [13, 15, 42], CD8^+^ T cells with effective cytotoxic activity against infection with Epstein-Barr virus (EBV) [24], and development of human B cells that produce antigen-specific IgM upon immunization with exogenous antigens [43, 44]. However, the extent of T and B cell maturation seems incomplete, leading to generation of cells that are not fully functional. For example, CD4^+^ T cells from humanized NOG mice responded poorly (compared with normal human T cells from healthy donors) to *in vitro *antigenic stimulation with anti-CD3 and anti-CD28 antibodies [45], and immunization of humanized mice with exogenous antigens was only able to induce a restricted immunoglobulin (Ig) G response [44–46].

HLA molecules are required for development of human T cells, and interactions between human B and T cells are essential to activate the molecular machinery responsible for B cell antibody class switching [47]. Thus, impaired human B and T cell function in humanized mice has been attributed to the absence of HLA in the mouse thymus [48]. In support of this notion, mice transplanted with human fetal liver and thymus under the kidney capsule and injected with HSCs (the bone marrow, liver, thymus (BLT) mouse model) had significantly improved human T and B cell function [49, 50]. Human HLA-DR (MHC class II equivalent) appears to play a more important role in T and B cell development and in T cell positive selection than HLA-A2 (MHC class I analog) in humanized mice. For example, while HLA-A2 transgenic mice elicit a slight improvement in human T cell reconstitution and function of T and B cells, HLA-DR4 or HLA-DR5 transgenic mice had significantly increased human cell reconstitution and better immune responses, including Ig class switching and elevated human IgG responses [51–54]. Furthermore, transgenic expression of human HLA-A2 in humanized NSG mice resulted in improved HLA-A2-restricted CD8^+^ T cell responses to both EBV and dengue virus infection [55–57]. It is noteworthy that in EBV-infected humanized HLA-A2 transgenic mice, T cell responses against lytic EBV antigens predominated over latent antigens, similar to what is observed in human EBV carriers [57].

## 4. *Ex Vivo* Expansion of Hematopoietic Stem Cells

It has become increasingly clear that developing better humanized mouse models will rely, at least in part, on increasing the quality of the HSC input material. Over the past few decades, the study of hematopoietic development and dynamic HSC interactions within the niche has shed light on signaling molecules that play roles in HSC self-renewal and lineage commitment. Rooted in this work, select cytokines and growth factors have been used to maintain and expand HSCs in culture, either alone or in combination [58]. Factors that have been used to promote expansion of human HSCs include Flt3 ligand [59], SCF [60–62], TPO [61, 63], IL-3 [64–66], IL-6 [65, 67], IL-11 [68, 69], and angiopoietin [70]. Although combinations of these cytokines and growth factors have been shown to promote *in vitro* proliferation of HSCs, the durability of this effect during short-term culture is limited as is the ability to maintain HSCs in an undifferentiated state. One explanation for this is the high sensitivity of HSCs to their microenvironment.

The role of cytokines, including IL-3 and IL-6, in the expansion of HSCs is somewhat controversial. On the one hand, there are reports of stimulatory effects on HSC *ex vivo* expansion and long-term repopulating capacity [64, 71, 72], whereas others have described an inhibitory effect [73, 74]. One strategy to address this has been to supply these factors by coculturing HSCs with stromal cells, although this technique has met with limited success [75], prompting investigation into other possible factors. Amongst these factors, pleiotrophin, the Notch receptor ligand Delta-1, angiopoietin-like protein 5 (Angptl5), hedgehog (Hh), p38 mitogen-activated protein kinase (MAPK) inhibition, prostaglandin E2 (PGE2), and StemRegenin (SR1) have been shown to stimulate human HSC proliferation and are further discussed below.

The neurite outgrowth factor pleiotrophin has been shown to promote *in vitro* expansion of both mouse and human HSCs [76]. This effect was observed on mouse bone marrow HSCs, where the protein caused a marked increase in numbers of long-term repopulating HSCs. Furthermore, treatment of human CB CD34^+^CD38^−^Lin^−^ cells with pleiotrophin in serum-free media containing TPO, SCF, and Flt3 ligands induced modest *ex vivo* expansion, but the fraction of CD34^+^CD38^−^Lin^−^cells was significantly higher, indicating a selective effect on differentiation rather than proliferation. Additionally, studies of transplantation using limiting dilution showed increased short- and long-term repopulating capacity of pleiotrophin-treated human CB CD34^+^CD38^−^Lin^−^ cells.

It has been proposed that the effect of pleiotrophin could be mediated by activation of phosphoinositide 3-kinase (PI3K)/AKT signaling in HSCs. This effect is most likely refereed by activation of Notch signaling, as antagonism of PI3K or Notch signaling pathways inhibits pleiotrophin-mediated expansion of HSCs in culture [76]. In fact, it is now widely appreciated that Notch signaling plays important roles in the regulation of proliferation and cell fate determination of HSCs. HSCs express Notch receptors, which bind to the transmembrane ligands Jagged-1, Jagged-2, and Delta [77]. Activation of these receptors on murine hematopoietic precursors by the immobilized extracellular domain of Delta1 fused to the Fc domain of human IgG1, [Delta1(ext-IgG)] resulted in marked proliferation of progenitors capable of short-term lymphoid and myeloid repopulation [67, 78]. Similarly, human HSCs cultured in serum-free conditions supplemented with SCF, Flt3-L, TPO, IL-3, and IL-6 were also responsive to activation by Notch ligands, and CD34^+^ cells underwent ~100-fold expansion in presence of Delta1 (ext-IgG) and exhibited enhanced repopulating ability in an immunodeficient mouse model [79, 80]. However, the effect on Notch signaling appears to be dependent on ligand abundance, since low-density Delta-1 enhances proliferation of CD34^+^ cells, whereas higher amounts induce apoptosis of CD34^+^ precursors [81].

One of the most pronounced amplifications of HSCs was achieved by supplementation with angiopoietin-like proteins (Angptls). These proteins were identified in mouse fetal liver CD3^+^ cells and may play a role *in vivo* in stimulating mouse fetal liver growth. In particular, Angptl2 and Angptl3 induced up to 30-fold expansion of long-term cultured mouse HSCs as determined by reconstitution analysis [82]. The same group found that another Angptl protein, Angptl5, acted more specifically on human HSCs and promoted ~20-fold net expansion of repopulating human CB HSCs when used in serum-free culture media containing SCF, TPO, fibroblast growth factor-1, and insulin-like growth factor binding protein 2 [83]. Although it appears that Angptls activate a distinct signaling pathway from other growth factors, the mechanism of action remains unclear.

The hedgehog (Hh) signaling pathway has been implicated in primitive and definitive hematopoiesis. In this regard, Bhardwajand and coworkers reported 60 to 80% increased proliferation of human CD34^+^CD38^−^Lin^−^ cells at 7 or 12 days in culture following addition of exogenous Hh in presence of SCF, G-CSF, Flt3 ligand, IL-3, and IL-6. Under these conditions, the cells retained their capacity to engraft into immunocompromised NOD-SCID mice [84]. The Hh pathway may also play a role in acute regeneration by inducing cell cycling and expansion of HSCs [85]. There is, however, controversial evidence regarding the role of Hh signaling in hematopoiesis. For example, studies targeting gain or loss of function reported no apparent effect on adult hematopoiesis [86, 87]. This discrepancy may be related to differences in experimental systems (e.g., human, mouse, or zebrafish), approaches (e.g., transgenic models, ES cells, or *in vitro* culture systems), genetic approaches to removing the Hh activator, smoothened (Smo), in Smo conditional knockout mice (i.e., use of different promoters to induce recombination), source of HSCs (e.g., fetal liver versus adult HSCs), and associated changes in developmental schedules.

Regarding regulation of HSC self-renewal, p38 MAPK has been identified as a key intrinsic negative factor. Activation of p38 MAPK has been associated with induction of HSC senescence under different physiological and pathological conditions [88, 89]. In line with these observations, selective inhibition of p38 MAPK activity with the synthetic agent SB203580 promoted *ex vivo* expansion of mouse bone marrow and human CB HSCs [90, 91]. Human umbilical CB CD133^+^ cells expanded in the presence of the drug by about threefold versus vehicle and displayed better engraftment into NOD-SCID mice following transplantation [91]. Improved self-renewal of HSCs following p38 MAPK inhibition has generally been attributed to inhibition of glycogen synthase kinase 3beta (GSK3*β*) and activation of the Wnt signaling pathway as evidenced by upregulation of the downstream target gene, HOXB4 [92].

Recent investigations seeking to identify modulators of HSC proliferation and homeostasis have revealed new targets. For example, prostaglandin E2 (PGE2) was identified in zebrafish by high-throughput screening of bioactive compounds regulating HSC expansion [93]. Receptors for PGE2 were found in mouse and human HSCs, and short-term *ex vivo* exposure of HSCs to PGE2 enhanced their homing to bone marrow via the chemokine receptor CXCR4 when transplanted into lethally irradiated hosts. These PGE2-treated cells also demonstrated increased proliferation, resulting in 4-fold increased long-term repopulating cell and competitive repopulating unit frequency, and enhanced survival associated with increased expression of Survivin [94]. Although its mechanism of action is not known, PGE2 has been shown to interact with the Wnt pathway by stabilizing *β*-catenin [95], highlighting the pivotal and beneficial role of Wnt signaling in HSC biology.

Another high-throughput screen, in this case of a drug library, revealed a purine derivative compound named StemRegenin 1 (SR1) that was able to promote *in vitro* expansion of mobilized human peripheral blood CD34^+^ cells in serum-free media containing TPO, IL-6, Flt3 ligand, and SCF [96]. The proliferative effect of SR1 on CD34^+^ cells did not occur in the absence of cytokines; it was reversible and had an anti-proliferative effect at high concentration, indicating that SR1 enhanced cytokine-mediated signals within a defined dose range. Interestingly, there were species-specific differences on SR1 bioactivity, as the compound did not expand murine HSCs, but potently affected human, monkey, and dog bone marrow-derived CD34^+^ cells. Furthermore, umbilical CB-derived CD34^+^ cells cultured for 3 weeks in the presence of SR1 had striking 17-fold expansion that improved early and long-term *in vivo* repopulation capacity in immunocompromised mice, and these cells retained multilineage potential [96]. The mechanism by which SR1 induces proliferation of HSCs is via direct binding to and antagonism of the aryl hydrocarbon receptor. 

Perhaps the most important among intrinsic factors are transcription factors, such as the members of the homeobox (HOX) gene family that have emerged as important regulators of hematopoietic cell proliferation and differentiation. In particular, HOXB4 and HOXA4 are potent *ex vivo* inductors of HSC expansion, as revealed by overexpression of these transcription factors in murine HSC culture experiments [97–99]. Human HSCs also appear to be responsive to HOX transcription factors. When cultured on stromal cells genetically engineered to secrete HOXB4, human long-term culture-initiating cells and NOD-SCID mouse repopulating cells expanded by more than 20- and 2.5-fold, respectively [100]. Likewise, HOXB4 overexpressing HSCs cells also displayed increased proliferation in culture [101]. In another report, Aurvray and coworkers showed that, in cocultures of HOXC4-producing stromal cells with human CD34^+^ HSCs, the HOXC4 homeoprotein expanded immature HSCs by 3 to 6 times in *in vitro* cloning assays and significantly improved *in vivo* engraftment in immunocompromised mice. Comparative transcriptome analyses of CD34^+^ cells subjected to HOXB4 or HOXC4 revealed that both homeoproteins regulated the same set of genes, indicating similar downstream effectors [102].

Wnt signaling is another pathway involved in development and function of HSCs. Forced expression of *β*-catenin enhanced *ex vivo* proliferation of murine HSCs by increasing HOXB4 and Notch-1 expression [103], and Wnt-3a induced self-renewal of mouse HSCs [104]. There is, however, controversial evidence from experiments based on constitutive activation of canonical Wnt signaling or *β*-catenin [105, 106]. Furthermore, no impairment of HSC function (e.g., self-renewal or reconstitution) was observed by inactivation of *β*- and *γ*-catenin [107, 108]. In experiments where the Wnt signaling pathway was modified by administration of GSK3*β* inhibitors or overexpression of Wnt5a, long-term repopulation was increased in mice transplanted with murine or human HSCs, but *in vitro* proliferation went unchanged [109, 110]. This controversy might be explained by differences in experimental systems or functional redundancy.

## 5. Inhibitory Signals and Control of Hematopoietic Stem Cell Expansion

**Figure 2:**
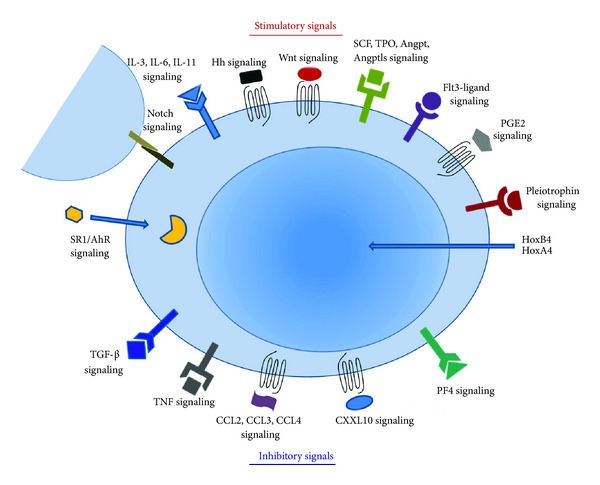
Diagram showing input from multiple signals that affect expansion of HSCs. Proliferation and differentiation of HSCs are regulated by the interplay of stimulatory and inhibitory modulators released by neighboring cells that inhabit the HSC niche within the bone marrow and other cell types within the hematolymphoid compartment. Synthetic molecules utilized to promote *ex vivo *expansion of HSCs are also depicted. Addition of stimulatory molecules combined with reduction in abundance of inhibitory factors represents a promising strategy to induce *in vitro *proliferation while maintaining HSCs in an undifferentiated state. Abbreviations used: AhR: aryl hydrocarbon receptor, Angpt: angiopoietin, Angptls: angiopoietin-like proteins, CCL: chemokine ligand, CXCL10: chemokine ligand 10, Hh: hedgehog, Hox: homeobox, IL: interleukin, PF4: platelet factor 4, PGE2: prostaglandin E2, SCF: stem cell factor, SR1: StemRegenin, TGF-*β*: transforming growth factor beta, TNF: tumor necrosis factor, TPO: thrombopoietin.

Most of the strategies designed to expand HSCs *in vitro* have focused on identifying molecules that promote self-renewal of the stem cell population. Comparably less attention has been directed toward inhibitory signals generated by the differentiated progeny and accumulated during the course of culture. Many of these factors, including transforming growth factor-*β* (TGF-*β*), tumor necrosis factor-alpha [111–113], and chemokines such as CCL2, CCL3, CCL4, and CXCL10 [114–116] have been reported to negatively impact the expansion of human hematopoietic stem and progenitor cells. Most of these inhibitory cytokines and chemokines are produced by monocytes and interact in an antagonistic manner with stimulatory factors from megakaryocyte origin (epidermal growth factor, platelet-derived growth factor subunit B, vascular endothelial growth factor, and serotonin) to modulate progenitor expansion [116]. In addition, highly purified bone marrow-derived CD34^+^ cells also secrete detectable amounts of growth factors, cytokines, and chemokines, which could affect their proliferation in an autocrine or paracrine manner by exerting either stimulatory (kit ligand, Flt3 ligand, and thrombopoietin) or inhibitory influences (TGF-*β*1, TGF-*β*2, and platelet factor 4) [117]. Various strategies for maintaining HSCs in culture are outlined in Figure 2.

Recently, Csaszar and colleagues described an integrated computational and experimental strategy to enable reduction of inhibitory signals in HSC culture media [118]. Based on the effect that feedback signaling from differentiated cells has on stem and progenitor cell expansion, the authors developed a fed-batch media dilution system, consisting of an input stream that results in a continuous increase in culture volume and, consequently, dilution of inhibitory signals. When compared to other media change protocols, including full media change every four days or every day, half media change twice a day, or continuous perfusion, the fed-batch system achieved the most effective enhancement in stem and progenitor expansion. Specifically, it yielded an 11-fold increase in HSCs from human cord blood after 12 days culture, and these cells demonstrated self-renewing, multilineage repopulating ability [118].

Several new factors and molecules have been utilized that, when combined with the most commonly used cocktail of cytokines, and have been successful to varying degrees at improving *in vitro* HSC expansion. However, at present, combination of these new molecules aiming to optimize culture conditions has not yet been reported. Thus, whether these new factors will have additive or even synergistic effects remains to be proven. Considering the potential for *in vitro* expansion and long-term engraftment in immunocompromised mice, the most effective factors for expansion of human HSCs to date are SR1 and Angptl5. They have shown similar ability to induce proliferation in culture while preventing differentiation and long-term engraftment in immunocompromised mice. 

## 6. Concluding Remarks

Efficient *ex vivo *expansion of HSCs still remains an elusive goal. The limited capacity of HSCs to self-renew in culture and their propensity to differentiate despite addition of cytokines and trophic factors represent significant hurdles that limit expansion and long-term engraftment potential of these cells. Nonetheless, significant advances have been made in this area. For example, the identification of new molecules that promote HSC proliferation and maintenance in culture and strategies designed to reduce the levels of inhibitors in the media represent important advances in this area.

With the creation of more sophisticated immunodeficient mice that exhibit improved reconstitution of the human hematolymphoid system, the application of HSCs to study human immune diseases in small animals has moved forward at a rapid pace. It deserves mentioning, however, that these models have limitations, such as the relatively short-term maintenance of chimerism and the poor reconstitution and function of T, B, and natural killer lymphocytes. The exogenous administration or endogenous expression of human growth factors or cytokines in these mice has improved both maintenance and reconstitution and holds future promise for the optimization of humanized mice.
